# Digital transformation of career landscapes in radiology: personal and professional implications

**DOI:** 10.3389/fradi.2023.1180699

**Published:** 2023-05-22

**Authors:** Anjali Agrawal

**Affiliations:** Teleradiology Solutions, Delhi, India

**Keywords:** teleradiology, artificial intelligence, emergency teleradiology, careers in radiology, work-life balance, digital transformation of radiology

## Abstract

Millennial radiology is marked by technical disruptions. Advances in internet, digital communications and computing technology, paved way for digitalized workflow orchestration of busy radiology departments. The COVID pandemic brought teleradiology to the forefront, highlighting its importance in maintaining continuity of radiological services, making it an integral component of the radiology practice. Increasing computing power and integrated multimodal data are driving incorporation of artificial intelligence at various stages of the radiology image and reporting cycle. These have and will continue to transform the career landscape in radiology, with more options for radiologists with varied interests and career goals. The ability to work from anywhere and anytime needs to be balanced with other aspects of life. Robust communication, internal and external collaboration, self-discipline, and self-motivation are key to achieving the desired balance while practicing radiology the unconventional way.

## Introduction

Digital transformation is the integration of digital technology into all areas of functioning, typically to drive efficiencies, growth and value (1). While it is certain that there will be digital transformation of health and healthcare, it is far less certain how such changes will impact health or healthcare, given loss of traditional inter-professional and doctor-patient relationships. Physicians have largely welcomed and championed such transformation of healthcare (2). Radiologists have been at the forefront of adopting newer technologies towards better patient care. Imaging being a naturally digital operation, and diagnostic radiology having less patient contact than most medical or surgical specialties, has led to important changes in how radiologists practice the profession and importantly, how they blend their personal and professional lives. While radiology has always been an attractive specialty for doctors seeking a good work-life balance, digital transformation has made it even more suitable and challenging by blurring the boundaries between work life and personal life (3–5). A three-dimensional view encompassing the practice of radiology, a perpetually connected life, and newer demands on radiologists helps in understanding the promises and perils of change.

I have been practicing teleradiology for nearly two decades with an international teleradiology group. I started relatively fresh out of a radiology residency program and a 1-year work experience at a hospital before embarking upon my teleradiology journey from across the other side of the globe with a chance to be at home and close to my family. This was all possible because of digital transformation of radiology, broadband penetration, and advances in information technology (6–9). Based on personal experiences over the last two decades as a teleradiologist, and with continuous digital evolution of our practice, I would like to highlight how technology has changed the career landscape in radiology and discuss ways to cope with these changes.

## Digitalization of radiology practice

### DICOM standard and PACS

#### Promises

The adoption of the DICOM standard enabled interoperability of imaging devices, making modern PACS possible. PACS, in turn, changed the face of radiology departments, both physically and culturally (6). For example, longitudinal assessment could be made efficiently at the convenience of a few clicks on a viewing monitor, in contrast to hunting for previous films in the now-extinct record rooms. Enterprise level PACS took it a step further, where clinicians were no longer compelled to visit the radiology department. Telephonic or electronic communications became sufficient for the most part. These efficiencies, which we all now take for granted, have occurred quite recently but have irreversibly transformed our practice.

#### Perils

Efficiency is just one parameter of quality. Face-to face communication has its own merits in terms of relationship building. The brief inadvertent hallway meetings and the physical clinic-radiological meetings provide interaction opportunities away from the humdrum routine work. Bonding with colleagues at physical meetings has immense personal and professional value. In an online forum, one has to try going beyond shop-talk to build a rapport of some sort. Conversation over caffeine breaks also allows for opportunities to bring up seemingly minor but possibly important topics, which otherwise get lost in brief structured interactions.

### Teleradiology

#### Promises

The digital nature of workflow has made it feasible for a radiologist to offer consultation from anywhere via teleradiology. This flexibility of location of work outside the confines of a designated radiology department or hospital has helped expand the radiologist workforce to outside the hospital, may be a different city or even another country (10). Intra-department teleradiology for after-hours and weekend and holiday coverage is being used by radiologists from their homes. The teleradiology concept was tested from across the coasts to utilize the different time zone advantage, by off- site radiology offices in lucrative locations such as Hawaii in the US, and then taken to another continent to ensure the radiologists can cover during their comfortable awake hours. This allowed international radiologists, like myself, trained in the US to return to their home countries and stay productive and maintain an uninterrupted service. It was a win-win situation as this minority of radiologists trained in different programs, not only contributed to the practices where they trained, but also added value and variety to local practices and academic programs. This diversity of experiences enriched the clinical practices and academic programs bringing in an international flavor and a wider perspective. Work from a place of one's choice is a boon for those who are primary care-takers of small children or ageing parents, which can be satisfying, both professionally and personally (11). Staying in a metropolitan city with particularly challenging traffic, work from home may result in huge time and energy savings, which one may channelize towards academic or recreational activities.

#### Perils

Staying real in a virtual environment requires conscious effort, unlike the traditional departments where a lot is absorbed by osmosis. Active engagement with colleagues during work hours is difficult in a teleradiology environment as besides being a virtual setting, the work is fast-paced and high volume, with turn-around-time being an important quality metric. Such an environment is usually devoid of traditional patient communication, which may lead to loss of sight of the bigger goal, which is an ailing patient behind every study. One is dependent solely on the history taking and examination skills of the referring physician. Security and confidentiality of patient data as it is transferred across the internet could be a challenge (12). Teleradiology service providers have to work harder to dispel the prevalent impression of being more prone to quality deficiencies due to varied locations of radiologists and virtual workflow.

#### What helped me

A balanced use of off-read-out time towards continuing medical eduction and vacation or a workation plays an important role in skill development and bonding with family and friends. After-hours meets with colleagues, if in the same city, or taking the initiative to meet a colleague visiting one's area, pays huge dividends in terms of providing a semblance of the real traditional office environment and sharing of problems which might be common or may be successfully resolved by communication. Actively contributing to professional groups is a great way to stay engaged and motivated. My regular attendance at the American Society of Emergency Radiology meetings provided a fertile ground for the founding of the Society for Emergency Radiology in India, and the connections across the globe offered ideas for solutions to local problems and a strong framework for collaborations, which have been successful for various academic activities.

Direct patient communication is challenging in a teleradiology environment, except for a unique second opinion service where the contractual agreement may be direct between the patient and the radiologist providing a second opinion. A discussion over the phone or on a virtual platform with the referring physician may help get a better sense of a complex situation or help convey the level of confidence of a radiologist for a particular pathology, increasing the acceptability of a teleradiology consultation amongst the referrers (13). I have found these phone calls with the physician extremely useful in formulating a more definitive impression such as in equivocal appendicitis on CT or for detecting a rib fracture in a CT with a limited written history of trauma and pain.

Secure transfer protocols for images and clinical information need to be well established by the radiology groups engaged in internal teleradiology or external teleradiology providers. A strong information technology support team is essential to this operation. Reliable and adequate internet is critical for a timely image transmission and communication, both verbal and sending the written report (12).

While teleradiology is not the same as work from home, the opportunity to work from home is tempting for many radiologists. This needs a proper mindset and physical structuring to be successful for the long haul. To be able to function effectively, segregation of professional and personal work is highly important. If possible, a designated space should be used for office work, with noise reducing partitions. The housework should be planned in a manner to avoid overlap with office hours, with a similar understanding with family members, while reassuring them of one's availability for any emergencies or crisis.

Quality assurance must be actively performed to ensure a seamless teleradiology practice and attention needs to be paid to all steps from image generation to transfer, reception, download, interpretation and report generation. These are taken seriously by external teleradiology providers with an aim to provide equal or better quality in terms of accuracy and timeliness. Regular systems maintenance are done remotely for teleradiologists by the IT department, all errors are discussed at the internal meetings at regular intervals to provide learning points for all, stakeholders are apprised of the newer features of the PACS-RIS. Sharing of the group's quality assurance initiatives and performance data helped me dispel the notion of substandard work by commercial teleradiologists (14–16).

## The perpetually connected digital life

Broadband penetration and mobile technologies have resulted in a digitally connected society that extends far beyond radiology. Radiologists are as susceptible to the stresses that it brings as other people, perhaps even more when it comes to physical problems associate with sitting for long times and staring at screens. It is not surprising therefore that health afflictions have increased in radiologists in recent years (17–20).

### Promises

There are many advantages to a connected life. Uninterrupted patient care with easy connectivity to healthcare providers is now considered routine. This is of course much easier for radiologists because of a natively digital workflow compared to other branches. With broadband penetration becoming ubiquitous, including during flights, 24/7 radiology is literally anytime anywhere.

### Perils

24/7 connectivity has blurred the boundaries between work and personal time. The expectations from radiologists are for a round the clock availability to support the medical and surgical specialties. One may still be required to work or attend to a business meeting while on vacation, which may lead to a feeling of not being able to do justice to either.

### What helped me

While, as a teleradiologist, it is entirely possible to carry my office with me and set up dual screens inside hotel rooms to work, I have steadfastly refused to be tempted. To me, it is good to know that if required in an emergency, one can briefly pitch in even during off-times, but it is not good to make it the norm. While each person should find what works best for them, it is likely that most people will do better by appropriate use of downtime to smell the not-digital roses.

## Working at superhuman capacity

The combination of digital workflows and perpetual connectivity is driving a new generation of radiologists to stretch beyond their comfort levels. Sustaining such levels of outputs risks burnout, if we do not find other ways of offsetting the increased load (17). Radiologist shortages, newer sophisticated imaging techniques and imaging protocols, and increasing expectations from referring doctors and patients have paved the way for deployment of artificial intelligence tools in different steps of the radiology workflow to make the processes more efficient and accurate with less human effort (21).

### Promises

Artificial intelligence is a technology disruption with a huge promise to increase radiologist efficiency to sustainably superhuman levels. What was perceived as a threat a few years ago, might be the radiologist's ally (22, 23). In addition to speed of diagnosis, certain AI tools have demonstrated accuracy higher than an average radiologist (24). Studies have shown improved results of AI assisted image interpretation over those by human alone (25). As of now, AI is a narrow intelligence useful for a specific task such as detection, localization and quantification of intracranial hemorrhage. When encountered with torso examinations with many more structures and pathologies to evaluate, a battery of AI tools would be required to provide a comprehensive interpretation. AI is certainly much faster, can work tirelessly and in certain tasks for long, thus, overcoming the limitations of a human radiologist. The AI tools can provide accurate volumetrics and objective measurements freeing up the radiologist from the mundane task of lesion measurements and allowing time for formulating a meaningful and actionable conclusion, leading to increased satisfaction (26–29). By taking over the boring and repetitive tasks, these tools may help reduce burnout amongst radiologists (17, 18). This also opens up newer avenues for research in AI in radiology, and career pathways in radiology focused on informatics and computer science.

### Perils

Workflow integration of AI has its own challenges, requiring an understanding of the technology, the tools that one plans to use and the background methodology used for development. This has its own learning curve akin to any other technology, posing a newer set of challenges in the daily workflow, temporarily, making it more laborious (30). Synthesis of AI outputs from multiple tools with one's core knowledge would become essential for one to stay on top of the field. Adapting to the AI enabled workflows would become essential for a successful radiology practice (28, 29).

### What helped me

Staying tuned with literature and expert talks on broad developments in AI was helpful in developing a sense of where the field is headed. I volunteered to participate in AI related research projects, investing time in the taskforce meetings, tool development and presentation of data at scientific forums for feedback and critique.

## Constants in changing times

Irrespective of the various changes being brought to radiology, and life in general, by digital transformation, some critical things remain constant.

First, communication is critical. Unlike face-to-face meetings, virtual meetings tend to be more task focused. Virtual socials may help in building relations, but they do not eliminate or undermine the importance of a face-to-face meeting. An opportunity to physically meet up with a virtual colleague should be cherished as these brief meetings can leave lasting memories and seal associations built in a virtual work environment. I am conscious of the importance of soft skills particularly in the current times of progressively increasing automation and do not shy away from presentations, teaching sessions, or spending an extra minute to communicate with the referring physicians.

Second, there has to be a balance between life and work. While for some, work may be life, the majority need off-time to stay sharp. Mental health issues are a major problem amongst radiologists (17) Taking care of body and mind is a critical aspect that is easy to neglect in fast moving digital lives.

Third, staying engaged within the workplace makes for a more fulfilling and satisfying work experience. This happens relatively easily at physical offices but needs extra effort when virtual. Something as simple as diligently blocking off time and participating fully in virtual meetings is helpful (31). Trying to multi-task during such meetings, sometimes being part of multiple virtual meetings, is a common digital-age problem that did not exist earlier. Staying real in a virtual environment requires discipline and self-motivation.

Last, but not least, these disruptions pose a new set of challenges, and in their wake are great opportunities. The ones who seize these opportunities and rapidly adapt can lead the field. A great example is my own group headed by a radiologist (32), who while a Yale faculty, studied the feasibility of international teleradiology for the United States from India as a project, which formed the basis for one of the largest teleradiology groups with a client base spanning the globe (8, 9). To stay abreast with the newer innovations and trends, and stay commercially viable, the group actively developed the technology to support its operations- an in house PACS-RIS and research in AI tool development and validation for improved efficiencies. There are many instances of unconventional radiology entrepreneurs and career paths revolving around AI in radiology, where there is a huge potential for making a greater impact. These newer avenues are aggressively being explored by professionals from other medical specialties as well as engineering and sciences backgrounds (33, 34). While collaborations become critical at this juncture, radiologists assuming leadership roles will be important for a brighter future of the field.

To conclude, the digital transformation of career landscapes in radiology is inevitable. Only part of the transformation is technological or radiology-related. This is the easier part that we, as natively digital doctors, understand well. The larger challenges are in navigating new times, when it is increasingly easy to be connected online, while becoming progressively more challenging to stay connected offline.
